# Genome-Wide Survey and Expression Analysis of *Chlamydomonas reinhardtii* U-box E3 Ubiquitin Ligases (CrPUBs) Reveal a Functional Lipid Metabolism Module

**DOI:** 10.1371/journal.pone.0122600

**Published:** 2015-03-30

**Authors:** Qiulan Luo, Yajun Li, Wenquan Wang, Xiaowen Fei, Xiaodong Deng

**Affiliations:** 1 Key Laboratory of Tropical Crop Biotechnology, Ministry of Agriculture, Institute of Tropical Bioscience and Biotechnology, Chinese Academy of Tropical Agricultural Science, Haikou, 571101, China; 2 School of Science, Hainan Medical College, Haikou, 571101, China; Mount Allison University, CANADA

## Abstract

E3 ubiquitin ligases determine the substrate specificity of ubiquitination. Plant U-box (PUB) E3 ligases, with a typical 70-amino acid U-box domain, participate in plant developmental processes and environmental responses. Thus far, 64 PUB proteins have been identified in *Arabidopsis* and 77 PUB proteins have been identified in *Oryza*. However, detailed studies on U-box genes in the model microalgae *Chlamydomonas reinhardtii* are lacking. Here, we present a comprehensive analysis of the genes encoding U-box family proteins in *C*. *reinhardtii*. Following BLASTP analysis, 30 full-length U-box genes were identified in the *C*. *reinhardtii* genome sequence. Bioinformatics analyses of CrPUB genes were performed to characterize the phylogenetic relationships, chromosomal locations and gene structures of each member. The 30 identified CrPUB proteins are clustered into 3 distinct subfamilies, and the genes for these proteins are unevenly distributed among 14 chromosomes. Furthermore, the quantitative real-time RT-PCR or semi-quantitative RT-PCR analysis of 30 CrPUB mRNA abundances under nitrogen starvation showed that 18 CrPUB genes were induced by N starvation and that 7 genes were repressed in the N-poor environment. We selected five CrPUB genes exhibiting marked changes in expression under N-free conditions for further analysis in RNAi experiments and examined the oil content of these gene-silenced transgenic strains. The silencing of CrPUB5 and CrPUB14, which are typically down-regulated under N starvation, induced 9.8%-45.0% and 14.4%-61.8% lipid accumulation, respectively. In contrast, the silencing of CrPUB11, CrPUB23 and CrPUB28, which are markedly up-regulated under N-free conditions, decreased the lipid content by 5.5%-27.8%, 8.1%-27.3% and 6.6%-27.9%, respectively. These results provide a useful reference for the identification and functional analysis of this gene family and fundamental information for microalgae lipid metabolism research.

## Introduction

Ubiquitin (Ub)-mediated protein degradation is a highly conserved process in eukaryotes. Ub, which is a small 76-amino acid protein, forms a multi-Ub chain that serves as a degradation tag. The covalent attachment of Ub polymers to specific proteins involves the following three-step reaction: 1) E1 (Ub-activating enzyme) consumes ATP and actives Ub; 2) activated Ub is transferred to E2 (Ub-conjugating enzyme), forming the E2-Ub intermediate; and 3) E2 interacts with E3 (ubiquitin-protein ligases), which binds the substrate and delivers Ub to the target [1]. The E3 ligase-mediated recruitment of substrates is the key to the specificity of ubiquitination. Thus, E3 ligases are a diverse gene family in plants. Plant E3s participate in signaling pathways and control the cell cycle, morphogenesis, stress responses, self-incompatibility and pathogen defenses [2,3]. In *Arabidopsis thaliana*, more than 1,400 different E3s genes have been predicted through comprehensive genome analysis [4,5].

Based on the subunit composition and action mechanism of E3s, these enzymes have been classified as single or multi-subunit ligases. The HECT and RING/U-box compose single-subunit ligases. The U-box protein was initially identified as an E4 ligase, the prototype of which is the yeast protein UFD2 [6]. UFD2 is required for the degradation of certain types of substrates, including a fusion protein with an NH2-terminal ubiquitin moiety. The UFD2 protein and its homologs in other eukaryotes share a conserved domain designated the ‘U box’. The U-box domain is a conserved 70-amino acid modified RING-finger domain that lacks the zinc-chelating cysteine and histidine residues that characterize most RING-finger domains [7]. Salt bridges and hydrogen bonds maintain the structure of U-box proteins. The U-box domain is directly relevant to the ubiquitin activity of U-box proteins. During ubiquitination, the U-box domain brings the E2 ligase in proximity to the E3-bound target for ubiquitination [8]; mutations in the U-box domain suppress ubiquitination [9]. Intriguingly, the database search revealed significantly more U-box protein-encoding genes in plant genomes compared with eukaryotic species. *Arabidopsis* and *Oryza* genomes contain 64 and 77 predicted plant U-box (PUB) members, respectively, whereas the yeast and human genomes contain only two and 21 U-box proteins, respectively [10,11]. The expansion of the plant U-box gene family suggests that these genes play diverse roles in cellular processes specific to plants.

Thus far, many studies examining the function of PUB E3 ligases have been reported. PUB E3 proteins have been implicated in many cellular processes, including seedling establishment, plant hormone responses, flowering, cell death, and abiotic and biotic stresses. Some upstream or target proteins of these ligases have also been identified (Table 1). The first characterized plant U-box E3 ubiquitin ligase was AtCHIP, which contains three additional tetratricopeptide repeats (TPRs). AtCHIP is responsible for the ubiquitination of protein phosphatase 2A (PP2A) and of the chloroplast proteins FtSH1 and ClpP4, which are involved in temperature stress and ABA responses and protein quality control in chloroplasts [12–14]. Plant U-box E3 ubiquitin ligases have multiple physiological functions in cells. For example, AtPUB13 and its *Oryza* ortholog protein SPOTTED LEAF11 (SPL11) both play important roles in spontaneous cell death (PCD), the salicylic acid (SA) response, biotrophic pathogen defense, and flowering time regulation [9,15,16]. In recent years, most PUB E3 ligases have been implicated in biological processes associated with abiotic stress responses. Loss-of-function and over-expression experiments have demonstrated that plant PUB ligases are either ABA-dependent or ABA-independent. AtPUB22/23 are negative regulators of drought responses, and this function is unaffected by ABA [17]. In contrast, AtPUB18/19, SAUL1/AtPUB43/44 and AtPUB9 are ABA-induced genes encoding plant U-box armadillo (Arm) repeat ubiquitin ligases. AtPUB18 and AtPUB19 are antagonistic proteins that act as negative regulators of ABA-mediated stomatal closure and water stress responses [18]. PUB44/SAUL1 has been implicated in leaf senescence via the negative regulation of ABA levels through AAO3, which catalyzes the last step in ABA biosynthesis [19]. In addition, several PUB proteins, such as AtPUB17, AtPUB20/21, NtACRE276, and NtCMPG1, have been implicated in plant pathogen defense [20–22]. Furthermore, many studies have shown that PUB proteins respond to plant growth hormones. For example, BnARC1 is necessary for the brassinosteroid (BR) self-incompatibility (SI) response [23], potato (*Solanum tuberosum*) PHOR1 protein is a positive regulator of gibberellin (GA) signaling [24], AtPUB4 controls the developmental fates of tapetal cells to ensure male fertility in parallel with the BR pathway [25], and OsTUD1 interacts with OsD1 to regulate BR-mediated growth [26]. Additional functions of PUB E3 ligases include nodulation in *Lotus japonicas* and *Medicago truncatula* (CERBERUS and MtPUB1, respectively [27,28]). Moreover, some PUB proteins participate in the nutrition starvation response: OsUPS responds to *Oryza* phosphate starvation [29], and AtPUB9, which was initially implicated in the ABA-mediated inhibition of seed germination, interacts with AtARK2 to form a signaling module (ARK2-PUB9) necessary for auxin-mediated lateral root development under phosphate starvation [30,31]. However, the function of PUB E3 ligases in many other plant species remains unclear, and the precise mechanism underlying the role of these proteins in physical responses is elusive. Specifically, little is known about the function of PUB proteins in microalgae.

**Table 1:** Known PUB E3 ubiquitin ligases and targets of the Ub/26S proteasome pathway involved in plant growth and development.

U-box E3 ligase	Motif containing	Regulatory pathway	Interacted protein	Reference
AtCHIP	U-box/TPRs	Temperature stress; ABA responses; chloroplast protein degradation	FtsH1, PP2A, ClpP4	[12–14,66]
AtPUB12/13	U-box/ARM	PCD and SA-defense; flowering signaling	AtFLS2	[9,16]
OsSPL11	U-box/ARM	PCD and SA-defense; flowering signaling	SPINs	[9,15]
PUB22/PUB23, PUB24	U-box	Pathogen response; negative regulators of drought responses	RPN12a	[17,18]
AtPUB18/19	U-box/ARM	Seed germination; ABA response; negative regulators of drought responses	unknown	[18,67,68]
AtPUB9	U-box/ARM	ABA signaling and drought; phosphate starvation response	ARK1	[30,31]
AtPUB43, AtSAUL1/44	U-box/ARM	Cell death; senescence control; seed germination and early seedling growth	AAO3	[69]
OsUPS	U-box/GKL	Phosphate starvation response	unknown	[29]
AtPUB17, NtACRE276	U-box/ARM	Pathogen response; cell death	unknown	[20]
AtPUB20/21, NtCMPG1	U-box/ARM	Pathogen response	AGB1	[21,22]
StPHOR1	U-box/ARM	light and GA signalling	unknown	[24]
BnARC1	U-box/ARM	self-incompatibility response	SRK, Exo70A1	[23]
AtPUB4	U-box/ARM	BR signaling pathway	unknown	[25]
OsTUD1	U-box	BR signaling pathway	D1/RGA1	[26]
CERBERUS	U-box/WDR	nodules development	unknown	[28]
MtPUB1	U-box/ARM	negatively regulates infection and nodulation	LYK3	[27]
OsPUB15/16	U-box/ARM	ROS stress; cell death	unknown	[70]
NtPUB4	U-box/ARM	self-incompatibility	CHRK1	[71]
CaPUB1	U-box	abiotic stresses response	RPN6	[56]

Microalgae have recently gained much attention as a potential source of renewable biodiesel production [32]; however, the knowledge concerning the biological mechanism of lipid metabolism in these organisms is limited. Stress conditions, particularly nitrogen limitation, induce triacylglycerol (TAG) accumulation in algae. The effects of nitrogen starvation on *C*. *reinhardtii* gene expression and metabolism have been studied for decades [33–35]. The fatty acid profile analysis showed a marked increase in saturated (palmitic acid; stearic acid) and monounsaturated (palmitoleic acid; oleic acid) fatty acids under nitrogen-deficient conditions. C16/C18 fatty acid accumulation in plants after cultivation under N-starvation for 3 days could reach up to 90.29% of total fatty acids compared to the 74.23% of total fatty acids detected on day 0 under N-starvation [36]. The results of studies measuring the changes in mRNA and protein abundances in *C*. *reinhardtii* under N starvation have generated conflicting results. The abundances of mRNA and proteins involved in carbon assimilation (Calvin-Benson cycle enzymes, acetate uptake and chlorophyll biosynthesis), photosynthetic complexes, and ribosomes in N-starved cells were reduced, and similar trends in the RNA abundance of putative transcription factor and transcriptional regulator genes were observed following N starvation [33]. However, N starvation activated a series of genes involved in gametogenesis [37]. The proteins involved in nitrogen assimilation, amino acid metabolism, oxidative phosphorylation, glycolysis, and the TCA cycle were elevated under N-starvation compared with non-stressed cultures [38]. In addition, the proteins associated with the formation of oil bodies in *C*. *reinhardtii* under N starvation were primarily involved in metabolism, transport, vesicle trafficking, and redox [34]. Additionally, the enzymes for lipid metabolism were significantly altered. The proteins and mRNAs encoding components involved in early fatty acid biosynthesis (ACCase, ACP, and FAS) were transiently repressed under N starvation but recovered to the N-replete level between 12 and 24 h after transfer to N-free medium [39]. The enzymes that mediate lipid biosynthesis, such as acyltransferases specific for TAG biosynthesis and three glycerol-3-phosphate dehydrogenase isozymes, showed consistent increased expression under N-deprived conditions, indicating short-chain free fatty acid accumulation [40]. The functional analysis of lipid synthesis enzymes has become a research hotspot. Many genes associated with triacylglycerol accumulation, such as DGAT, PDAT and GPDH, have been characterized [41–43]. However, the involvement of E3 ubiquitin ligases in *C*. *reinhardtii* lipid metabolism remains unclear.

Ubiquitin was identified in *C*. *reinhardtii* as early as 1990 in response to heat shock and photoinhibition [44]. The ubiquitin system has been implicated in many processes throughout the *C*. *reinhardtii* cell, including circadian clock control, cilia and flagella disassembly, the abiotic stress response and environmental signal transmission [45,46]. Using comparative genomics, the sequences of genes encoding E3 ligases in *C*. *reinhardtii* have been compared with other eukaryotic ubiquitin ligase genes [47]. In the present study, we performed a homologous sequence search in the *C*. *reinhardtii* genome and proteome to obtain a preliminary understanding of CrPUB E3 ubiquitin ligases. In addition, we analyzed the mRNA abundance of CrPUB genes under nitrogen starvation through real-time quantitative or semi-quantitative RT-PCR. The expression of CrPUB genes markedly changed under nitrogen starvation, and these genes were selected for further analysis through RNA interference. The lipid content in RNA interference transformants was examined. Taken together, these results suggest an important role for CrPUB E3 ubiquitin ligases in lipid metabolism in *C*. *reinhardtii*.

## Materials and Methods

### Identification of U-box-containing proteins in *C*. *reinhardtii*

The latest version (V5.5) of the *C*. *reinhardtii* genome and protein sequences was downloaded from the Phytozome V10.0 database (http://phytozome.jgi.doe.gov/pz/portal.html). BLASTP analysis was performed using the published plant U-box proteins (*Arabidopsis* and *Oryza* PUB proteins) as query sequences, with an E-value cutoff of 1e-005. Only non-redundant full-length sequences containing more than 60 residues were considered, and these CrPUB protein sequences were identified through PFAM (http://pfam.sanger.ac.uk) and SMART (http://smart.embl-heidelberg.de/). Moreover, conserved protein motifs were predicted using PROSITE (http://prosite.expasy.org/) and MEME (http://meme.nbcr.net/meme/). The molecular weights and isoelectric points of proteins were predicted using Expasy (http://web.expasy.org/compute_pi/). The potential sub-cellular locations of these CrPUB proteins were predicted using WoLF PSORT [48].

### Phylogenetic analysis

Multiple sequence alignments were generated using ClustalX 2.1, and the alignments were edited using the GeneDoc 2.7 sequence editor. Maximum-likelihood (ML) trees were constructed using PhyML (approximate likelihood ratios analysis) [49]. Mega6 was used to generate neighbor-joining (NJ) trees [50]. In total, 1000 bootstrap replicates were performed to establish the reliability of the NJ and ML trees. The evolutionary tree diagrams were edited using FigTree 1.41 software (http://tree.bio.ed.ac.uk/software/figtree/). The gene clusters were generated based on the results of the alignments.

### Sequence properties and chromosomal locations

The structures of CrPUB genes were generated online using the Gene Structure Display Server (GSDS) (http://gsds.cbi.pku.edu.cn/). The protein motif annotation was performed using the SMART program. The duplication patterns of the U-box genes were analyzed based on their locations in the *C*. *reinhardtii* genome. The starting position of each CrPUB gene was obtained from the *Chlamydomonas* sequencing database. The locations of 30 CrPUB genes were drafted using MapInspect software (http://www.plantbreeding.wur.nl/uk/software_mapinspect.html). The candidate CrPUB genes were shown from the top to the bottom on *C*. *reinhardtii* chromosomes according to their positions. The homologous chromosome segments were detected using a synteny plot in Plaza (http://bioinformatics.psb.ugent.be/plaza/versions/pico-plaza/synteny/index). The CrPUB genes were subjected to BLAST analysis in Plaza, and their duplication patterns were detected using a synteny plot.

### Microalgae strains and culture conditions

The *C*. *reinhardtii* strain CC124 was used in nitrogen starvation experiments. The strains were incubated under continuous illumination (180 μmol m^-2^ s^-1^) on an orbital shaker (220 rpm) at 24°C with standard CO_2_ levels. The cells were initially cultured photoautotrophically to the mid-logarithmic phase in high-salt (HSM) medium. These pre-cultured cells were collected by centrifugation and resuspended at a density of 0.5–1.0×10^6^ cells/mL in HSM lacking nitrogen (HSM-N). For transformation, the *C*. *reinhardtii* cell wall defect strain CC425 was grown in Tris/acetate/phosphate (TAP) medium under the same conditions.

### Gene expression analysis

Three independent populations of 4-day-old cells grown in HSM or HSM-N were collected. The cells were frozen in liquid nitrogen, and total RNA was isolated using TRIzol (Invitrogen). Total RNA was treated with DNAse and purified using an RNeasy Mini Kit (Qiagen). cDNA was synthesized using a PrimeScript Double Strand cDNA Synthesis Kit (TaKaRa) according to the manufacturer’s instructions. Target gene expression patterns were measured by real-time quantitative PCR using an Agilent StrataGene Mx3005. The PCR reactions was performed using a SYBR Premix Ex Taq Kit (Takara), and the PCR conditions included denaturation at 95°C for 5 min, followed by 35 cycles of denaturation at 95°C for 1 min, annealing at 60°C for 30 s, and extension at 72°C for 20 s. 18S rRNA was used to normalize the expression ratio. The primers used in the present study are listed in S1 Table. Semi-quantitative RT-PCR was performed in a final volume of 20 μL containing 2 μL of diluted cDNA, 10 μL of 2X Premix Taq Mix version 2.0 (TaKaRa), and 200 nM of forward and reverse primers (S1 Table).

### RNAi experiments

The genes significantly down- or up-regulated by N starvation were selected for further analysis using RNA interference experiments. Two sets of RNAi primers for each gene were used to generate CrPUB gene-specific dsRNA. The primer sequences are shown in S2 Table. Primer set A was applied for amplifying the fragments from the non-conservative domain-encoding region, and primer set B was applied for randomized design. The pMaa7IR/E3sIR vectors were constructed as described previously [51]. For transformation, *C*. *reinhardtii* CC425 was grown in TAP medium to a cell density of 1–2×10^6^ cells/mL. The cells were collected and resuspended at a cell density of 1×10^8^ cells/mL. The transformation was performed using glass beads with 2 μg of plasmid DNA. To facilitate the induction of RNAi, the cells were plated on selective media containing 1.5 mM L-tryptophan, 5 μg/mL paromomycin and 5 μM 5-FI after recovering for 1 day. The resistant strains were tested by quantitative PCR to verify the suppression of mRNA expression.

### Lipid content assay

To determine the lipid contents of the RNAi transformants, the Nile red fluorescence method was applied according to Chen [52]. Briefly, the cells were resuspended in 200 μL of staining solution containing 25% (v/v) DMSO and 0.5 μg mL^-1^ Nile red dye for 10 min, and then fluorescence detection (FD) was performed using a Glomax-Multi Detection System (Promega), with excitation and emission wavelengths of 530 nm and 575 nm, respectively. Triolein (Sigma) was used as the lipid standard. The cell density (numbers/L) was determined using a cell counting method. The lipid content (ng/10^6^ cells) was calculated using the following equation: [0.0004×FD(530/575)-0.0038]×0.05/cell numbers.

For microscopic analyses, the cells were stained with Nile red (10 g/m^3^ final concentration), and the images were acquired using a Nikon 80i fluorescence microscope. Nile red signals were captured at an excitation wavelength of 480 nm, and the emission was collected between 560 and 600 nm.

### Statistical analyses

The data are presented as the means±S.D. One-way analysis of variance (ANOVA), followed by Duncan’s post-test, was used to examine significant differences between the means. In all cases, comparisons showing a p value<0.05 were considered significant.

## Results

### Characterization of CrPUB proteins in *C*. *reinhardtii*

To identify the U-box proteins in *C*. *reinhardtii*, we first collected the known PUB gene sequences from *Arabidopsis thaliana* (AtPUB1-AtPUB64) and *Oryza sativa* (OsPUB1-OsPUB77) and then performed a BLASTP search in the *C*. *reinhardtii* V5.5 proteome database using these sequences as queries. SMART and Pfam analyses were performed to remove putative pseudogenes and incorrectly annotated genes, resulting in the identification of 30 members through either SMART (SM00504) or Pfam (PF04564). The 30 non-redundant U-box genes in *C*. *reinhardtii* were named CrPUB1-CrPUB30. The features of the 30 CrPUB genes, including the gene locus, chromosome position, open reading frame (ORF) and amino acid lengths, protein predicted molecular weights and isoelectric points (pIs) are listed in Table 2. The encoded CrPUBs varied from 207 to 4072 amino acids (aa) in length, with an average of 1100 aa. The isoelectric points of the 30 CrPUB genes ranged from 4.40 (CrPUB27) to 9.40 (CrPUB14). The CrPUB sub-cellular locations were predicted using the WoLF PSORT program. CrPUBs were distributed throughout the entire cell, primarily in the chloroplasts and nuclei.

**Table 2:** List of the 30 U-box genes identified in *C*. *reinhardtii* and their sequence characteristics.

Gene symbol	Gene locus	Chromosome position^a^	ORF (bp)	Amino Acids/kD	pI	Sub cellular location^b^
CrPUB1	Cre01.g016800	1:2821083..2825144F	2259	752/76.7	6.87	N
CrPUB2	Cre03.g144344	3:213700..224555R	4113	1370/140.4	5.67	V
CrPUB3	Cre03.g178550	3:4792961..4796760R	1392	463/50.7	6.54	c
CrPUB4	Cre03.g185750	3:5501325..5504240R	891	296/31.7	5.47	C
CrPUB5	Cre05.g234654	5:2206384..2212259R	2619	872/87.0	5.52	N
CrPUB6	Cre06.g277300	6:3309571..3316540R	1839	612/59.5	8.72	C
CrPUB7	Cre08.g383550	8:4644495..4652226F	1599	532/56.8	6.07	N
CrPUB8	Cre09.g389450	9:2727631..2739456F	6726	2241/218.9	5.98	N
CrPUB9	Cre09.g390356	9:3637478..3644653R	3759	1252/130.3	7.78	c
CrPUB10	Cre09.g392505	9:3947811..3954082F	3723	1240/124.0	5.10	N
CrPUB11	Cre09.g398350	9:1446312..1454418R	3156	1051/115.0	5.53	C
CrPUB12	Cre09.g399550	9:1239632..1245623F	1584	527/54.7	6.41	C
CrPUB13	Cre10.g417850	10:50016..58677F	6207	2068/204.9	6.44	C
CrPUB14	Cre10.g443700	10:3326469..3336890F	5457	1818/180.4	9.40	c
CrPUB15	Cre10.g443950	10:3357640..3362478R	1524	507/53.1	8.97	E
CrPUB16	Cre10.g444100	10:3380497..3383262R	1542	513/53.8	8.78	c
CrPUB17	Cre10.g449200	10:4026895..4042865R	12225	4072/393.1	6.02	N
CrPUB18	Cre10.g454300	10:4848918..4855739F	3150	1049/ 106.3	6.30	n.a
CrPUB19	Cre11.g479650	11:3234241..3238105R	804	207/29.7	6.35	N
CrPUB20	Cre12.g507200	12:2401258..2407789F	2745	914/91.5	8.34	M
CrPUB21	Cre12.g519150	12:4241958..4250690R	4317	1438/147.6	6.11	c
CrPUB22	Cre12.g532050	12:5608063..5614238R	2790	929/95.3	6.88	N
CrPUB23	Cre12.g549250	12:8357638..8361403R	1464	487/49.4	6.71	C
CrPUB24	Cre13.g576550	13:1960588..1964402F	1089	362/37.9	9.37	C
CrPUB25	Cre13.g604501	13:4691805..4703296F	9651	3216/304.9	5.87	N
CrPUB26	Cre14.g612850	14:755536..761727F	3303	1100/106.5	4.84	C
CrPUB27	Cre15.g635750	15:318207..328642R	2472	823/85.7	4.40	C
CrPUB28	Cre16.g689250	16:3753670..3766021F	3435	1144/117.7	5.63	C
CrPUB29	Cre17.g705450	17:1270499..1274026F	1719	572/59.8	6.38	c
CrPUB30	Cre17.g743847	17:6646013..6652056F	1683	560/59.3	5.37	c

^a^F and R represent the forward and reverse directions on the chromosome, respectively.

^b^WoLF PSORT. N, nucleus; C, chloroplast; c, cytoplasm; V, vacuole; E, endoplasmic reticulum; M, mitochondria; n.a., not available.

In total, 30 CrPUB proteins were obtained by BLASTP search using the *C*. *reinhardtii* V5.5 proteome database and PUB proteins from *Arabidopsis thaliana* and *Oryza sativa* as queries. The 30 CrPUB genes were named based on their chromosome position. The molecular weights and pIs of the 30 CrPUB proteins were predicted using ExPASy. The CrPUB sub-cellular locations were predicted using the WOLF PSORT program.

### Domain organization of the CrPUB E3 ligases

**Fig 1:**
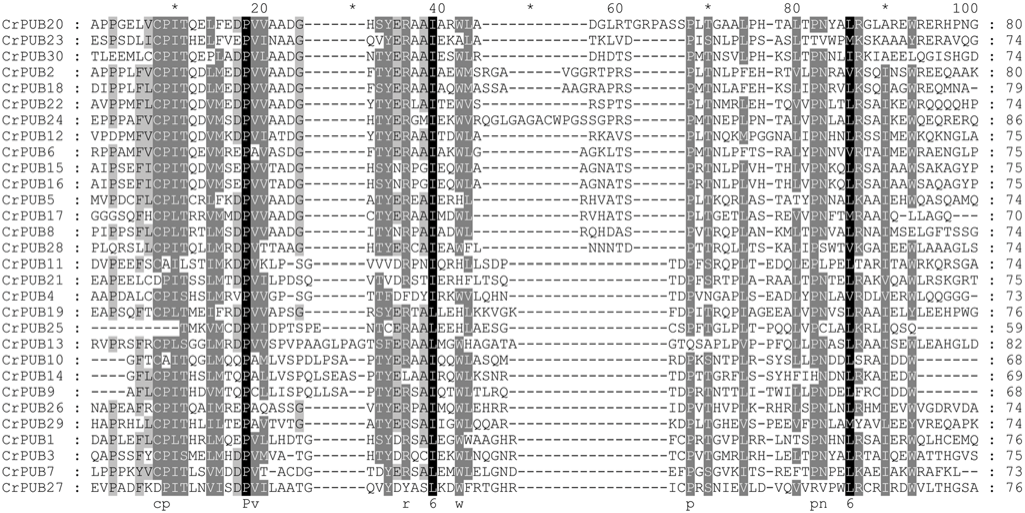
Multiple alignment of U-box domains from CrPUB proteins. The U-box domains in CrPUB proteins were predicted using PROSITE and MEME programs. Their sequences were aligned using ClustalX 2.1, and the alignments were edited using the GeneDoc 2.7 sequence editor. Black, gray and light gray shading indicates the identities and similarities among these sequences as 100%, 80%, and 60%, respectively.

**Fig 2:**
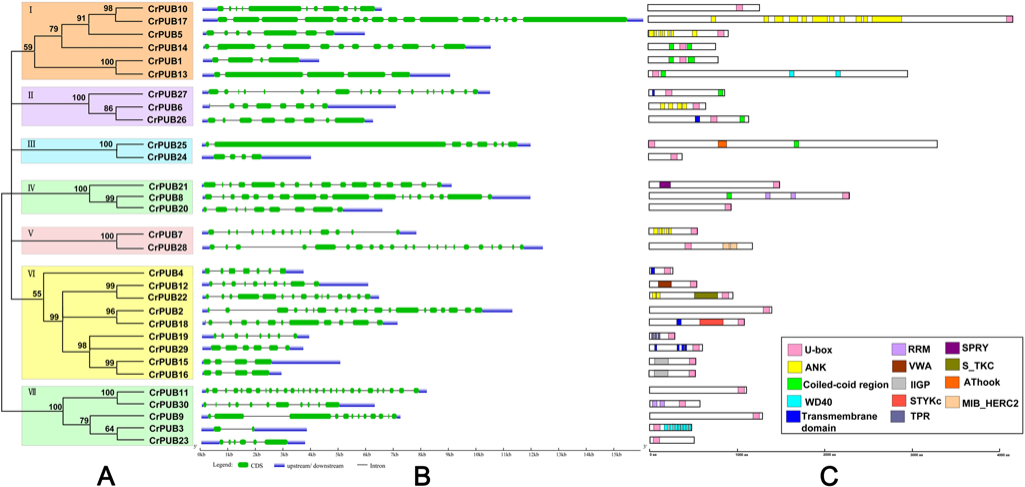
An analytical view of the CrPUB gene family. A. An unrooted tree summarizing the evolutionary relationships among the 30 members of the CrPUB family. Multiple alignments of the 30 PUB protein sequences from *C*. *reinhardtii* were conducted using ClustalX 2.1. The phylogenetic tree was constructed using PhyML3.01 and the ML method with 1,000 bootstrap replicates. The numbers on each node are Shimodaira-Hasegawa-like test indices of statistical support provided by PhyML. Bar = 2.0 is a branch length that represents the number of amino acid substitutions per site. The tree shows the 3 phylogenetic subfamilies (numbered I to III and marked with different color backgrounds) with high predictive values. B. Intron/exon structure: The gene structures were drawn using the online tool GSDS. As shown in the legend, the exons and introns are indicated by green rectangles and thin lines, respectively. The untranslated regions (UTRs) are indicated by blue boxes. The sizes of exons and introns can be estimated using the scale shown at the bottom. C. Schematic representation of the conserved motifs in the 30 CrPUB proteins elucidated using SMART and PROSITE online. The different domains are indicated by different colored boxes denoted at the bottom right corner. The lengths of the proteins and motifs can be estimated using the scale shown at the bottom.

Using PROSITE and MEME prediction, the 30 CrPUB E3 ligases were reconfirmed as U-box ubiquitin ligases. Multi-sequence alignment of the U-box motifs in the CrPUB proteins showed that the U-box domain is conserved in all 30 CrPUBs (Fig 1). Eukaryotic U-box and RING-finger proteins possess consensus sequences, including hydrophobic (LIYFWVMA) and aliphatic (LIVAM) sequences. In U-box proteins, the metal-chelating residues have been replaced partially or completely in the conserved RING-finger domains [53]. The critical conserved amino acid residues in the U-box domains of CrPUBs are PLIVM (Fig 1). The average length of the U-box domains in CrPUBs is 74 aa, with the shortest being a 59-aa motif in CrPUB25 and the longest being an 86-aa motif in CrPUB24. In addition to the U-box, various other protein domains/motifs are present in CrPUB E3 ubiquitin ligases (Fig 2C). In total, 13 conserved additional domains, except the U-box domain, were predicted in CrPUB proteins, such as WD40, TPR, ankyrin, RRM, VWA, IIGP, STKc, SPRY, STYKc, AThook, MiB_HERCI, coiled-coil region and transmembrane motifs (Fig 2C). Most of the additional domains of CrPUB proteins harbor coiled-coil regions and an ankyrin repeat domain. The numbers of CrPUB ligases containing coiled-coil regions, Ank repeats and a U-box domain are 7, 5 and 7, respectively.

### Classification of U-box proteins in *C*. *reinhardtii*

We constructed an evolutionary tree of the CrPUB proteins using PhyML with the ML method. According to the phylogenetic analysis, the 30 CrPUB proteins were divided into 3 subfamilies, designated I, II and III (Fig 2A). Although the proteins with low bootstrap values were not classified into subfamilies, these proteins were considered in additional analyses. The only U-box domain-containing protein assemblies in subfamily I contained proteins with U-box domains at the C-terminus. Most subfamily I proteins also contained transmembrane domains. The subfamily II genes are the longest in the CrPUB family, encoding proteins with the highest average amount of amino acids. The U-box motifs in subfamily II proteins are primarily located at the N-terminus and in the middle of the proteins, except CrPUB8 and CrPUB17 (Fig 2C). The CrPUB proteins possessing ankyrin repeat domains were not divided into a subfamily primarily because of the number of domains and because the amino acids conjugating the domains are extremely various (Fig 2).

### Gene structure and gene duplication of CrPUB genes

**Fig 3:**
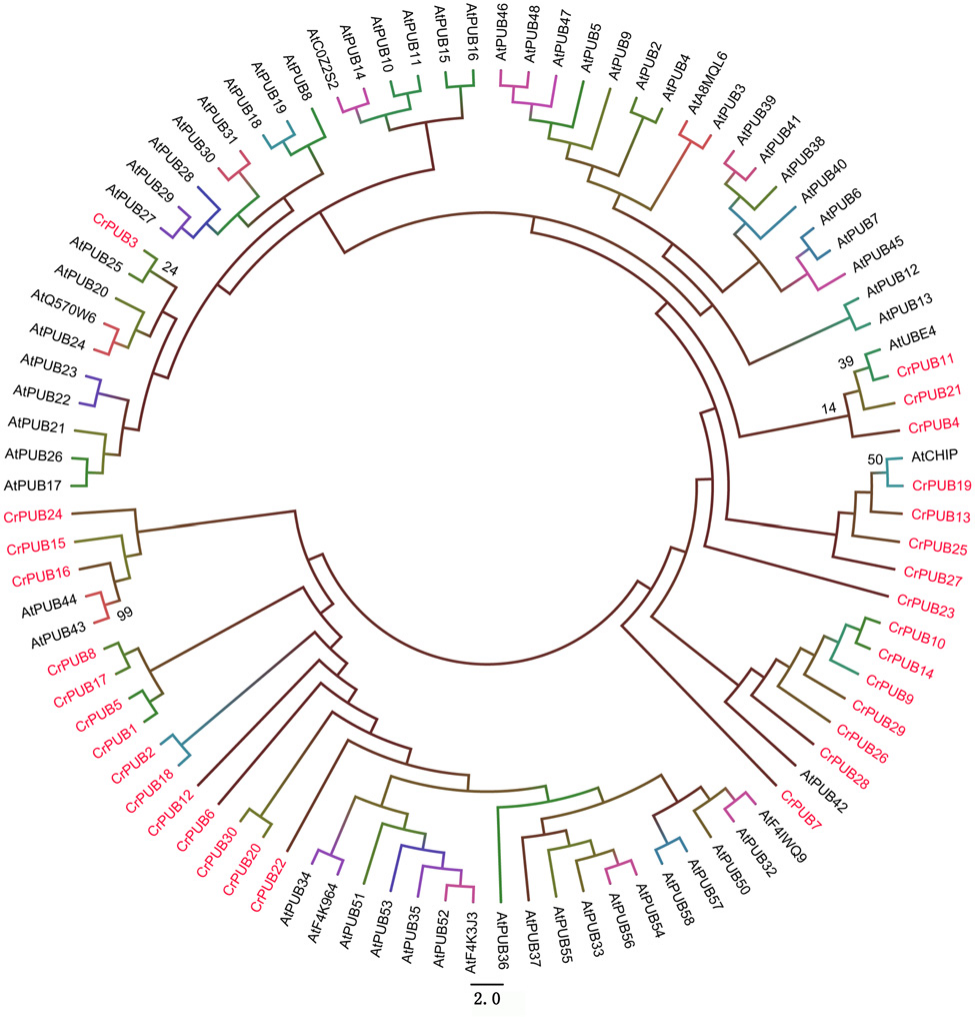
Phylogenetic relationship between *C*. *reinhardtii* and *Arabidopsis* U-box proteins. The amino acid sequences of U-box proteins from the two proteomes were used for analysis. The unrooted tree was inferred using Mega 6.0 software and the neighbor-joining method with 1000 bootstrap replicates. The CrPUB proteins are indicated in pink font; only the *Arabidopsis thaliana* and *C*. *reinhardtii* homologous branches show the bootstrap values as percentages.

To obtain further insight into the structural diversity of CrPUB genes, the genomic structures of the CrPUB genes, including the numbers and lengths of introns/exons, were analyzed using GSDS (Fig 2B). According to the intron/exon architectures of the CrPUB genes, multiple introns exist in these genes; even CrPUB19, which is composed of the least number of amino acids, contains 7 introns. The protein with the least number of introns is CrPUB3, which is most homologous with the AtPUB proteins (Fig 3). The longest exon was observed in CrPUB25, with a length of 8.3 kb. Unfortunately, no obvious regularity in the intron/exon structures was found within the same subfamilies. The lengths and numbers of introns were markedly different between each subfamily (Fig 2). For example, in subfamily I, the number of introns varied from 5 to 27, and the length of the introns ranged from 250 bp to 1.5 kb.

**Fig 4:**
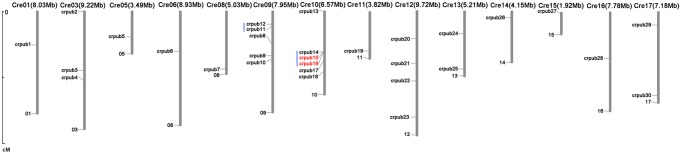
Chromosomal locations of the 30 CrPUB genes. The locations of the 30 CrPUB genes were drafted using MapInspect software, and their duplication patterns were detected using a synteny plot in Plaza. The chromosome numbers and sizes are shown at the top of each chromosome. Each CrPUB gene on the chromosome is displayed on the left side according to the approximate physical location. The tandem gene duplications are indicated in red. The blue line indicates a gene cluster.

According to the starting position of CrPUB genes on the chromosome, the 30 CrPUB genes were not evenly distributed among the 14 chromosomes, except for chromosomes 2, 4, and 7 (Fig 4). Chromosome 10 contains the most CrPUB genes (six), and five CrPUB genes are on chromosome 9. The longest chromosome (chromosome 12) in *C*. *reinhardtii* has four CrPUB genes. Three CrPUB genes were identified on chromosome 3, while two CrPUB genes were mapped to chromosomes 13 and 17. In contrast, a majority of chromosomes possess only one CrPUB gene, including chromosomes 1, 5, 6, 8, 11, 14, 15 and 16. In *C*. *reinhardtii*, only five CrPUB genes were identified in two gene clusters that were found on chromosomes 9 and 10 (Fig 4). The gene duplication events of these 30 CrPUB genes were analyzed using PLAZA software. Only two tandem repeat genes, CrPUB15 and CrPUB16, were observed in subgroup III in *C*. *reinhardtii*, which were deduced according to the genomic locations (Fig 4).

### Relationship between CrPUB and AtPUB proteins

The presence of a variety of additional domains in CrPUB proteins makes the grouping of these proteins more sophisticated than that for AtPUB and OsPUB proteins. To investigate the evolutionary relationships between the PUB proteins in *C*. *reinhardtii* and *Arabidopsis*, we performed a phylogenetic analysis of CrPUB and AtPUB proteins to construct a phylogenetic tree (Fig 3). First, the full-length PUB proteins were aligned using ClustalX 2.1, and subsequently, a phylogenetic tree was generated using the neighbor-joining method. For statistical reliability, we conducted bootstrap analysis with 1000 replicates. The phylogenetic tree showed that the CrPUB proteins are extremely different from AtPUB proteins (Fig 3); only CrPUB3 showed slight homology with AtPUBs. AtPUB42, 43, 44, AtCHIP and AtUBE4 showed some similarity with CrPUB proteins; however, the bootstrap values were low in these nodes. These results suggest that the CrPUB proteins markedly differ from the AtPUB proteins.

### The expression patterns of CrPUB genes under nitrogen starvation

**Fig 5:**
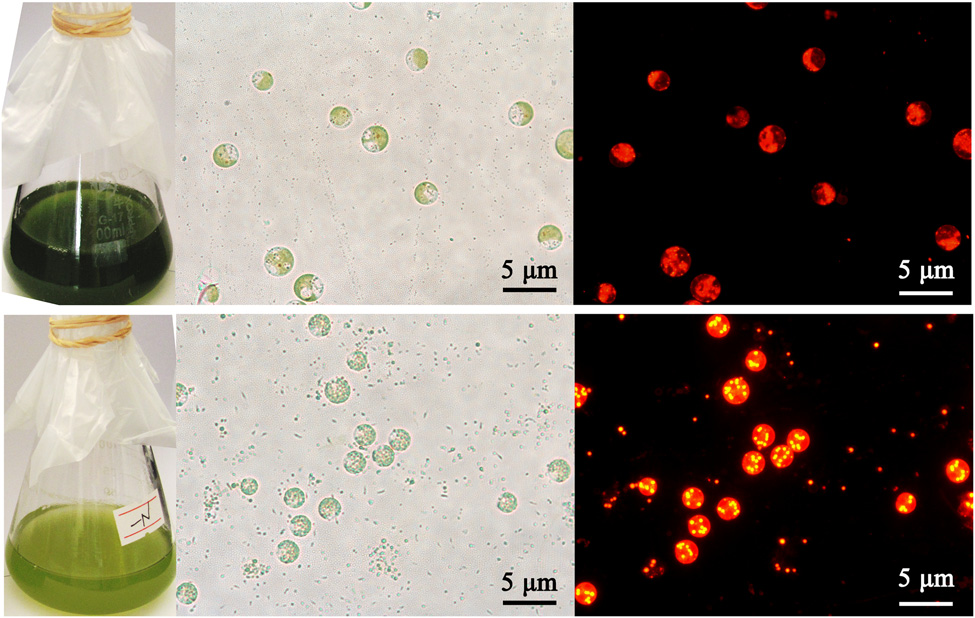
Effect of nitrogen starvation on lipid accumulation in *C*. *reinhardtii*. Microalgae cells were cultured in HSM and HSM-N for 4 days. The cell culture medium turned yellow when grown under N starvation. The nonpolar lipid in cells was stained using Nile red and imaged using a Nikon 80i fluorescence microscope. The lipid drops are shown as yellow dots under dark field. Red indicates chlorophyll autofluorescence. The upper panel shows cells grown in HSM, and the lower panel shows cells grown in HSM-N. From left to right: cell culture fluids, bright field images and fluorescent images. Scale bar = 5 μm.

**Fig 6:**
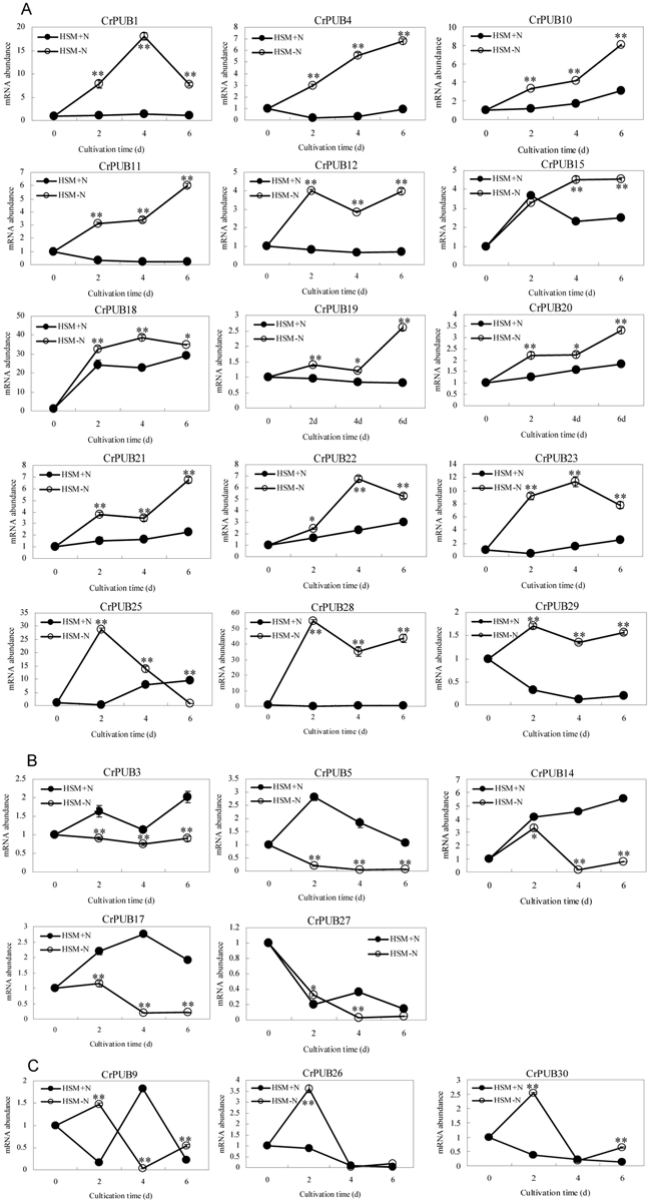
Results of qPCR analysis of the CrPUB genes. A. N starvation up-regulated the mRNA expression of 15 CrPUB genes as shown by real-time PCR analysis. B. The transcription of 5 CrPUB genes was repressed under N starvation. C. CrPUB9, CrPUB 26 and CrPUB30 were all expressed under both N starvation and normal conditions; however, no relationship between mRNA abundances and N starvation was found. *C*. *reinhardtii* CC124 were pre-cultured in HSM to the mid-logarithmic phase, followed by centrifugation and resuspension in HSM and HSM-N with continued culturing for 0, 2, 4, 6 days. The cells were collected, and the RNA samples were isolated. The gene transcript levels were determined using real-time quantitative PCR. All expression values were normalized to the value of the 18S rRNA gene. The relative amounts were calibrated based on the number of transcripts of the corresponding genes in cells maintained in HSM-N for 0 days. The data are shown as the means (±SD, n = 3). Significance is indicated as *P<0.05, **P<0.01. The hollow circles represent CrPUB gene mRNA abundances under N starvation, and the solid circles represent CrPUB gene mRNA abundances under normal conditions.

In *C*. *reinhardtii*, lipid droplets accumulate under nitrogen (N) starvation (Fig 5). Because the gene expression patterns often imply gene function, we examined the expression patterns of *C*. *reinhardtii* PUB genes in cells cultured in nitrogen starvation medium (HSM-N) for 0, 2, 4 and 6 days. The full-length cDNA sequences of CrPUB genes were obtained from Phytozome V10.0. Quantitative RT-PCR (qPCR) primers, which were designed using Primer Premier software version 5.0, are shown in S1 Table. The transcript abundances of CrPUB genes were analyzed using real-time quantitative PCR. As shown in Fig 6, 23 CrPUB genes were broadly expressed in *C*. *reinhardtii* maintained in both HSM and HSM-N for 0, 2, 4 and 6 days. In total, 15 up-regulated CrPUB genes were detected according to the qPCR results (Fig 6A). Among these genes, CrPUB18 and CrPUB28 dramatically increased several hundred times more than those detected in the cells grown under normal N conditions. In total, 8 CrPUB genes displayed induced expression in cells upon N depletion, and the transcript levels reached their highest values after N starvation for 6 days. CrPUB25, CrPUB28 and CrPUB29 expression was sensitive to N starvation; these genes were induced at 2 days under nitrogen starvation. CrPUB1, CrPUB18, CrPUB22 and CrPUB23 showed peaked transcription at 4 days following the removal of N from the nutrient solution (Fig 6A). In contrast, only 5 CrPUB genes, including CrPUB3, CrPUB5, CrPUB14, CrPUB17 and CrPUB27, were down-regulated under N starvation conditions (Fig 6B). The expression levels of these genes were lowest after 4 days under N starvation conditions. The analysis of the expression of CrPUB9, CrPUB26 and CrPUB30 genes showed that mRNA transcription was not affected under N starvation (Fig 6C).

**Fig 7:**
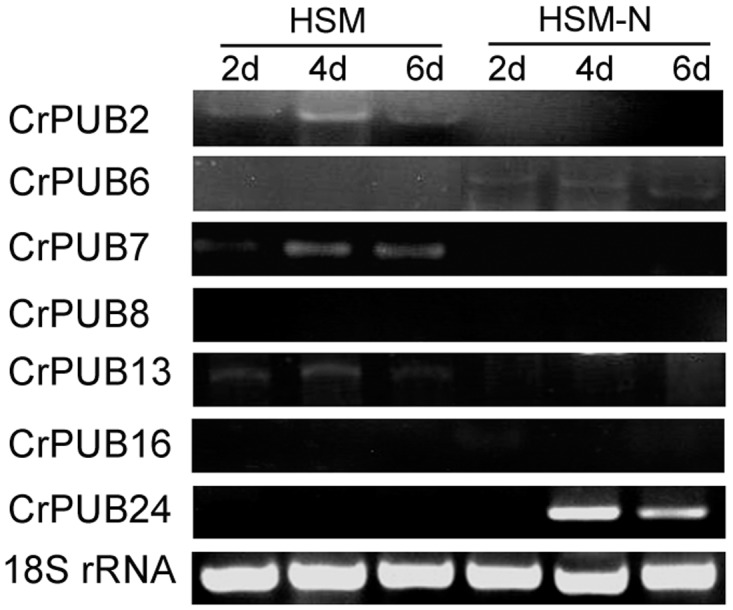
The expression patterns of CrPUB genes as determined by semi-quantitative PCR analyses. Microalgae cells were grown in complete (HSM) and N-starvation (HSM-N) media for 2, 4 and 6 days. Semi-quantitative PCR analyses were performed. The 18S rRNA gene was used as an internal control.

Because 7 CrPUB genes showed no CT values during real-time quantitative PCR, we performed semi-quantitative PCR using RNA isolated from cells grown in HSM and HSM-N for 2, 4 and 6 days (Fig 7). CrPUB8 and CrPUB16 showed no expression in either HSM or HSM-N. However, CrPUB2, CrPUB7 and CrPUB13 were expressed only when cultured in complete medium and displayed a relatively low level of expression. CrPUB6 showed weak expression in N-deficient medium and no expression in HSM. CrPUB24 expression was significantly induced under N starvation for 4 and 6 days, but no transcript was detected in HSM after 2 days under N starvation.

### RNAi-mediated silencing of CrPUB genes affects the oil content in *C*. *reinhardtii*

**Fig 8:**
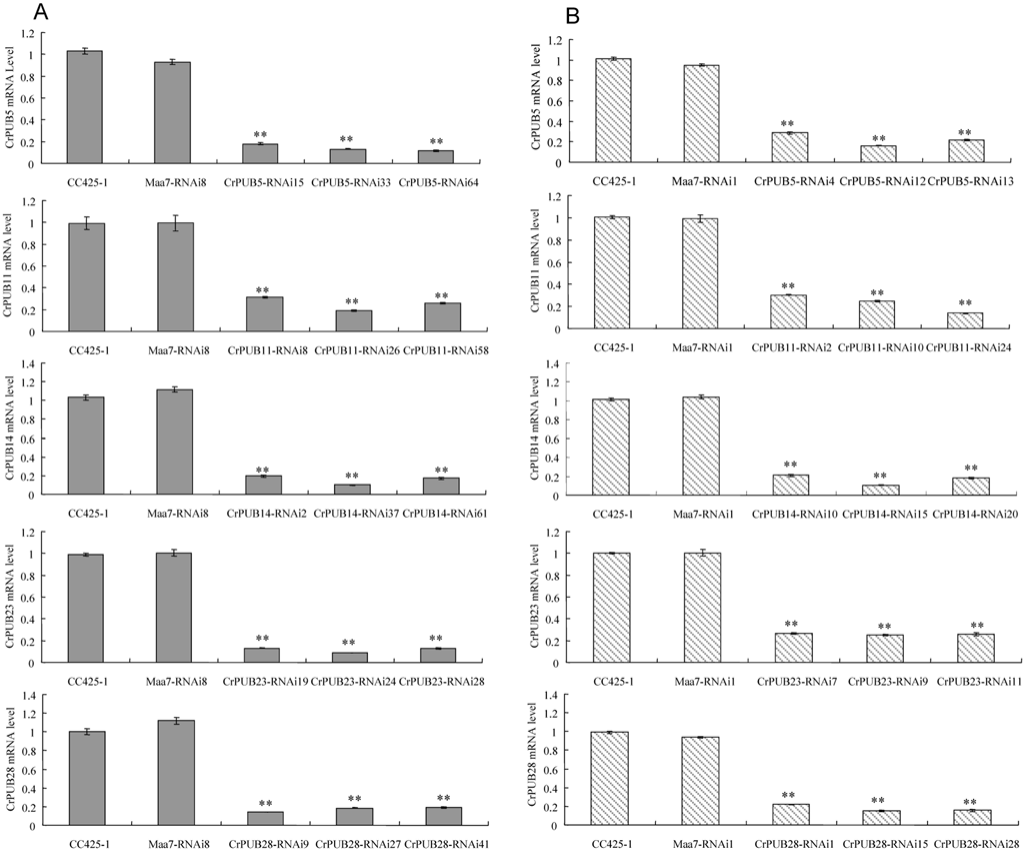
Comparison of the mRNA abundance of CrPUB genes in RNAi transgenic strains and wild type *C*. *reinhardtii* CC425. (A) CrPUB gene transcript levels in RNAi lines containing the constructs harboring the fragments amplified using primers set A as the dsRNA. (B) CrPUB gene transcript levels in RNAi lines containing the constructs harboring the fragments amplified using primers set B as the dsRNA. The dsRNA was obtained from RT-PCR amplification and subcloned into pMaa7IR/XIR. The constructs were transformed into CC425 using the glass bead method. In total, at least 30 resistant strains were selected in each case. CrPUB gene transcript levels were determined by real-time RT-PCR, with the 18S rRNA gene used as an internal control. The height of each column presents the relative gene expression compared with that in wild type CC425 strains. The examined gene transcription levels in empty-vector transformants are shown as Maa7-RNAi. The gene abundances in CrPUB5, CrPUB11, CrPUB14, CrPUB23 and CrPUB28 RNAi lines are shown from the top to bottom, respectively. Significant differences were observed between the RNAi lines and the controls (**P<0.01 Duncan’s post-test). The data are presented as the means of three replicates from one experiment. The error bars represent SD.

**Fig 9:**
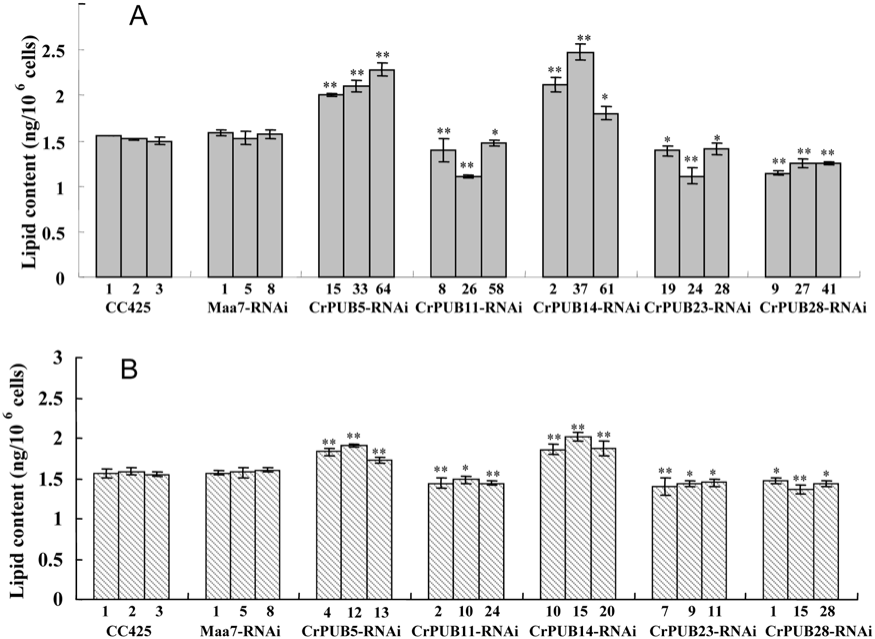
Lipid contents in CrPUB gene RNAi transgenic lines. (A)The lipid contents in the RNAi lines containing the constructs harboring the fragments amplified using primer set A as the dsRNA. (B) The lipid content in RNAi lines containing the constructs harboring the fragments amplified using primers set B as the dsRNA. The CrPUB gene RNAi lines were cultured in HSM for 12 days. The cells were resuspended in 200 μL of Nile red staining solution, followed by FD. The lipid content (ng/10^6^ cells) was calculated using the equation mentioned previously. Both the wild type CC425 and empty-vector transformants Maa7-RNAi were presented as controls. Significant differences were assessed using ANOVA. The p-values indicate the level of significance for differences between RNAi lines and wild type (*P<0.05, **P<0.01). The data are expressed as the means±SD of 3 replicates. CrPUB5 and CrPUB14 gene silencing induced lipid accumulation, and CrPUB11, CrPUB23 and CrPUB28 gene silencing showed the opposite effect.

**Fig 10:**
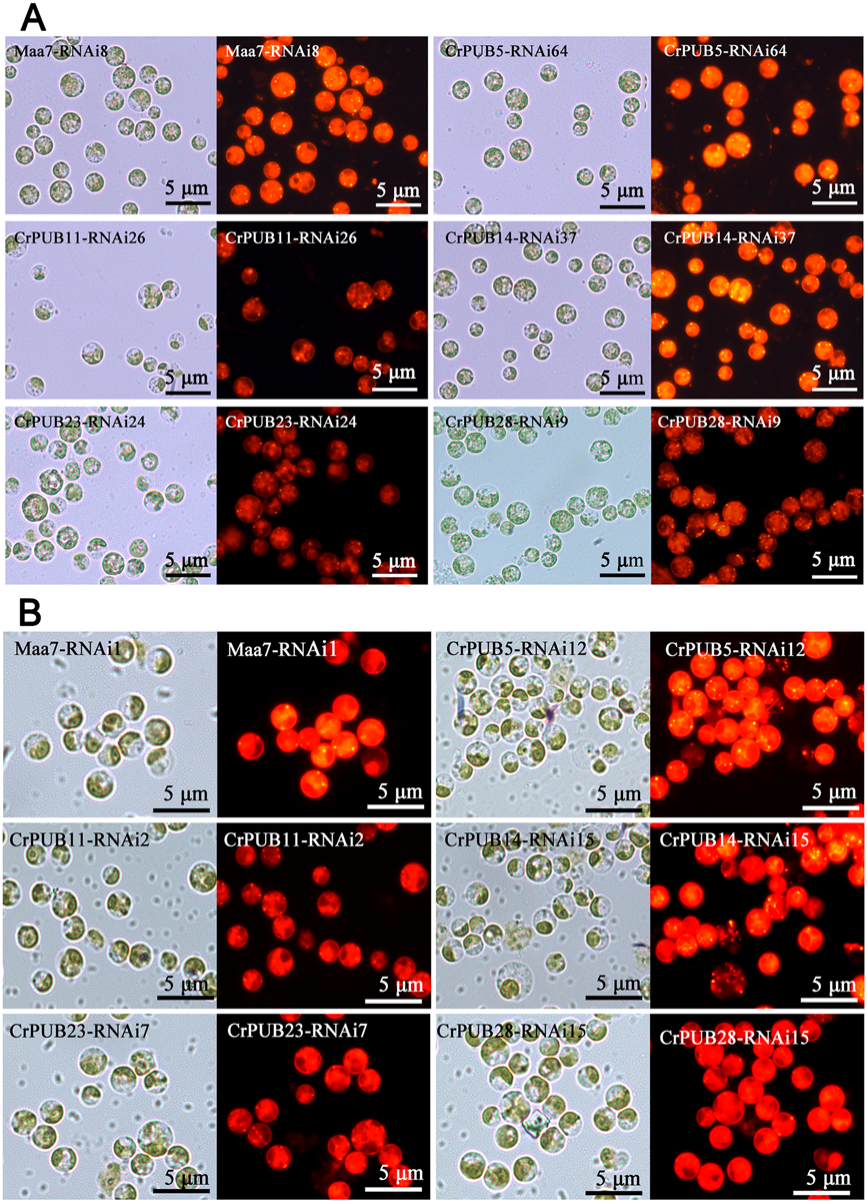
Microscopic observations of CrPUB gene RNAi transgenic algae. (A) Fluorescence microscopy for the detection of nonpolar lipid accumulation using Nile red staining of CrPUB RNAi lines containing the constructs harboring the fragments amplified using primer set A as the dsRNA. (B) Fluorescence microscopy for the detection of nonpolar lipid accumulation using Nile red staining of CrPUB RNAi lines containing the constructs harboring the fragments amplified using primer set B as the dsRNA. After culturing in HSM for 12 days, the cells of the RNAi lines were stained with Nile red and imaged using a Nikon 80i fluorescence microscope. Yellow fluorescence signals indicate lipid drops, while the red background indicates chlorophyll autofluorescence. Scale bars = 5 μm. Each picture represents a bright field image (left) and a fluorescent image (right).

Five CrPUB genes, including CrPUB5, -11, -14, -23 and -28, displayed dramatically affected mRNA expression under nitrogen starvation and were selected for additional analyses using RNAi experiments. The fragments were subcloned into the pMaa7IR/XIR vector in sense and antisense directions interrupted by the 18S intron under the CaMV35S promoter. The vector constructs were transformed into *C*. *reinhardtii* CC425 using the glass bead method. The cells were collected from at least 30 transgenic lines in each case, and their CrPUB gene transcript levels were determined by qRT-PCR. Moreover, we selected three highly suppressed lines in each CrPUB constitutive RNAi group (Fig 8). We also included three empty vector-transformed lines and wild type CC425 as controls. All transgenic lines and controls were cultivated in HSM for 12 days before measuring the oil content using the Nile red fluorescence method. In CrPUB5 and CrPUB14 RNAi lines, which were generated using primer set A, the lipid content increased by 26.5%-45.0% and 14.4%-61.8%, respectively (Fig 9A). In contrast, transgenic lines carrying the siRNA against CrPUB11, CrPUB23 and CrPUB28 exhibited decreased lipid contents. The oil contents of algae transformed with CrPUB11, CrPUB23 and CrPUB28 RNAi constructs decreased by 6.5%-27.8%, 10.5%-27.3% and 18.4%-27.9%, respectively (Fig 9A). We conducted the gene silencing experiments using another set of primers (set B) and obtained three RNA silencing lines for each CrPUB gene. Among them, CrPUB5 and CrPUB14 RNAi algae exhibited the highest lipid accumulation, showing approximately 10.0%-21.3% and 18.0%-28.4% higher accumulation, respectively, than that observed in control lines. The lipid contents of CrPUB11, CrPUB23 and CrPUB28 RNAi lines decreased by 5.5%-8.5%, 8.1%-11.5% and 6.6%-13.6%, respectively (Fig 9B). No obvious differences were found between the lipid contents of CrPUB RNAi lines generated using primer sets A and B (Fig 9). The observation that the RNAi-mediated silencing of the expression of five CrPUB genes affected the lipid content suggested that these five CrPUB genes are involved in the biosynthesis of lipids in *C*. *reinhardtii*. Similar results were obtained from the Nile red staining analysis; a few oil droplets with yellow florescence were detected in CrPUB11, CrPUB23 and CrPUB28 RNAi transgenic strains, and more oil droplets were observed in CrPUB5 and CrPUB14 RNAi transgenic algae (Fig 10A). The microscopy analysis of the RNAi lines obtained using primer set B showed reduced lipid synthesis in cells containing CrPUB11, CrPUB23 and CrPUB28 RNAi lines, while cells containing CrPUB5 and CrPUB14 RNAi lines showed oil accumulation (Fig 10B).

## Discussion

U-box proteins have been identified in all eukaryotic organisms, including fungi, plants and animals. Previous studies have shown that U-box proteins play a variety of important roles in many cellular processes [3]. Accumulating evidence suggests that plant U-box proteins (PUB) are critical for plant growth and development. The features and functions of the PUB gene family have been identified and analyzed in *Arabidopsis* and *Oryza* [10,25,54]. A genome-wide analysis of the PUB genes in the model microalgae *C*. *reinhardtii* would provide more information concerning this gene family. In the present study, 30 *C*. *reinhardtii* PUB proteins were identified and characterized in detail (Table 2). The number of U-box containing genes in *C*. *reinhardtii* was approximately less than half of that present in *Arabidopsis* and *Oryza* [10]. These findings may suggest that the number of PUB genes in the three species is associated with the total gene number because the gene number in *C*. *reinhardtii* is nearly half of the *Arabidopsis* gene number and one-third of the *Oryza* gene number [55].

All CrPUB proteins contain a complete and conserved U-box domain (Fig 1), which contributes to E3 ubiquitin ligase activities. Mutations in U-box motifs often disrupt the structure and function of these enzymes [9,56]. Furthermore, additional conserved domains have been identified in PUB proteins; these additional motifs also assume important functions in protein reactions. The classification of plant U-box E3 ligase is different from that of other gene families, and this classification is not based on gene homology but on the presence or organization of additional domains, except the U-box [54]. In *Oryza* and *Arabidopsis*, 8 and 7 groups of PUBs, with additional domains have been identified, respectively. These additional domains are as follows: ARM, STK, WDR, TPR, LRR, UFD2, GKL-box, and MIF4G [57]. *Arabidopsis* and *Oryza* PUB proteins possess the same types of PUB proteins, but CrPUB proteins possess different types and numbers of additional domains (Fig 2C), suggesting that U-box ligases in unicellular algae are involved in ubiquitin-dependent degradation, with specific activities compared with those in advanced plants. Based on the protein structures, an interesting phenomenon was observed, namely, the Arm-PUB subfamily, which contains the highest number of PUB family members in both *Arabidopsis* and *Oryza*, does not exist in *C*. *reinhardtii* [58]. The arm domain present in AtPUB and OsPUB is necessary for substrate conjugation during ubiquitination and plasma membrane association [59]. Arm repeat proteins also exist in *C*. *reinhardtii*, but have not been identified in the PUB protein family, suggesting that the functions of CrPUB proteins are irrelevant to the Arm repeat domain, unlike higher plant PUB proteins. However, the ankyrin repeat domains contained in the ANK protein family, which is another crucial superfamily in higher plants, have been observed frequently in the CrPUB family. Plant ANK proteins play major roles in the regulation of cell differentiation and development in response to disease resistance and stress [60–62]. In total, 5 CrPUB proteins harboring ANK repeat domains mediate ubiquitination. Whether this function relies on ANK repeat domains requires further studies. Furthermore, the proportion of CrPUB proteins only containing a U-box domain among the total number of U-box ligases in *C*. *reinhardtii* is higher than that in higher plants, suggesting that CrPUB proteins interact with substrates in a simpler manner.

Microalgae have been considered the original ancestors of plant in evolutionary analysis. In the present study, significant differences between CrPUB and AtPUB proteins were observed through phylogenetic analysis (Fig 3). These phenomena suggested that CrPUB proteins are more diverse in protein regulation functions than higher plant PUB proteins. In the present study, the 30 CrPUB proteins were divided into three subfamilies based on phylogenetic analysis but not according to the type of the additional motifs in the PUB proteins as reported previously [10] because the 30 CrPUB proteins contained 13 additional motifs and because some proteins possess more than two types of additional domains. Some genes were difficult to assign to any subfamilies because of the low bootstrap values in the clades. Interestingly, CrPUB3, the nearest related protein to AtPUBs, cannot be classified into any subfamilies in *C*. *reinhardtii*.

In the present study, the exon/intron structures of CrPUB genomic sequences were markedly different, even within the same subfamily. The diversity in the position and number of introns in CrPUB genes might be associated with the critical roles of CrPUB proteins in protein interactions and in regulating different cellular processes. In total, 30 CrPUB genes possessing two gene clusters were distributed among chromosomes 9 and 10, and one tandem gene duplication was also identified (Fig 4), which is far less than that detected in *Arabidopsis* and *Oryza*. This finding may be associated with the number of PUB genes present in the three species.

Previous studies have demonstrated that the plant U-box family is involved in nutrient defect responses, organ morphogenesis, stress responses and disease resistance in plants [18,31,63,64]. Nitrogen is an important nutrient element that influences lipid/carbohydrate accumulation in various species of microalgae [65]. In the present study, we identified 25 CrPUB genes involved in responses to nitrogen starvation through quantitative or semi-quantitative RT-PCR analysis. The results the gene expression analyses (Fig 6 and Fig 7) showed 18 CrPUB genes with significantly higher expression levels when the cells were maintained in N-deficient medium. In addition, 7 CrPUB genes showed inhibited expression under N starvation. U-box proteins were identified as E3 ligases involved in protein ubiquitination. The fact that most of the CrPUB genes promoted expression under N-free conditions may indicated that U-box proteins accumulate in *C*. *reinhardtii* to adjust metabolic pathways to adapt to the N-deficient environment. However, the 30 CrPUBs were divided into 3 subfamilies based on close evolutionary relationships, with different expression profiles in each subfamily. The different expression profiles suggest that these CrPUB genes participate in different molecular mechanisms involving host adaptation to complicated environmental challenges. Notably, CrPUB16 was not detected in any condition, while the highly homologous gene CrPUB15 showed induced transcript levels under N starvation. This finding may reflect the presence of CrPUB16 pseudogenes in *C*. *reinhardtii*.

One of the primary effects of nitrogen starvation on microalgae is that nitrogen starvation leads to oil accumulation compared with normal conditions (Fig 5). The expression of CrPUB genes was associated with nitrogen starvation conditions. However, the oil content was significantly affected in selected CrPUB RNAi expression transformants. Nitrogen starvation down-regulated CrPUB5 and CrPU14 expression but resulted in increased lipid synthesis when these genes were silenced, in contrast to the up-regulated CrPUB genes CrPUB11, CrPUB23 and CrPUB28 (Fig 9 and Fig 10). Taken together, these results suggested that CrPUB genes play roles in oil metabolism in cells. *C*. *reinhardtii* is an important model unicellular organism that is widely used in physiological, genetic and molecular studies of microalgae. The present study has identified and characterized the PUB genes in *C*. *reinhardtii*. These findings should provide important information concerning protein regulation in microalgae oil metabolism for guiding future experimental work on CrPUB genes in this model species.

## Supporting Information

S1 Table The primers of CrPUB genes used for real-time quantitative RT-PCR. (PDF)

S2 Table Primers sequences of the 5 selected CrPUB genes for RNAi expression analysis. A: The primers used for the amplification of RNAi expression fragments from non-conserved domain-encoding regions. B: Primers randomized in design for the amplification of RNAi expression fragments. The sequence, length, Tm, GC% and product sizes of the primers are shown in the table.
(PDF)
