# Correction: Genome-Wide Survey and Expression Analysis of *Chlamydomonas reinhardtii* U-box E3 Ubiquitin Ligases (CrPUBs) Reveal a Functional Lipid Metabolism Module

**DOI:** 10.1371/journal.pone.0142996

**Published:** 2015-11-11

**Authors:** Qiulan Luo, Yajun Li, Wenquan Wang, Xiaowen Fei, Xiaodong Deng

Fig 2 is incorrect in the published article. The authors have provided a corrected version here.

**Fig 2:**
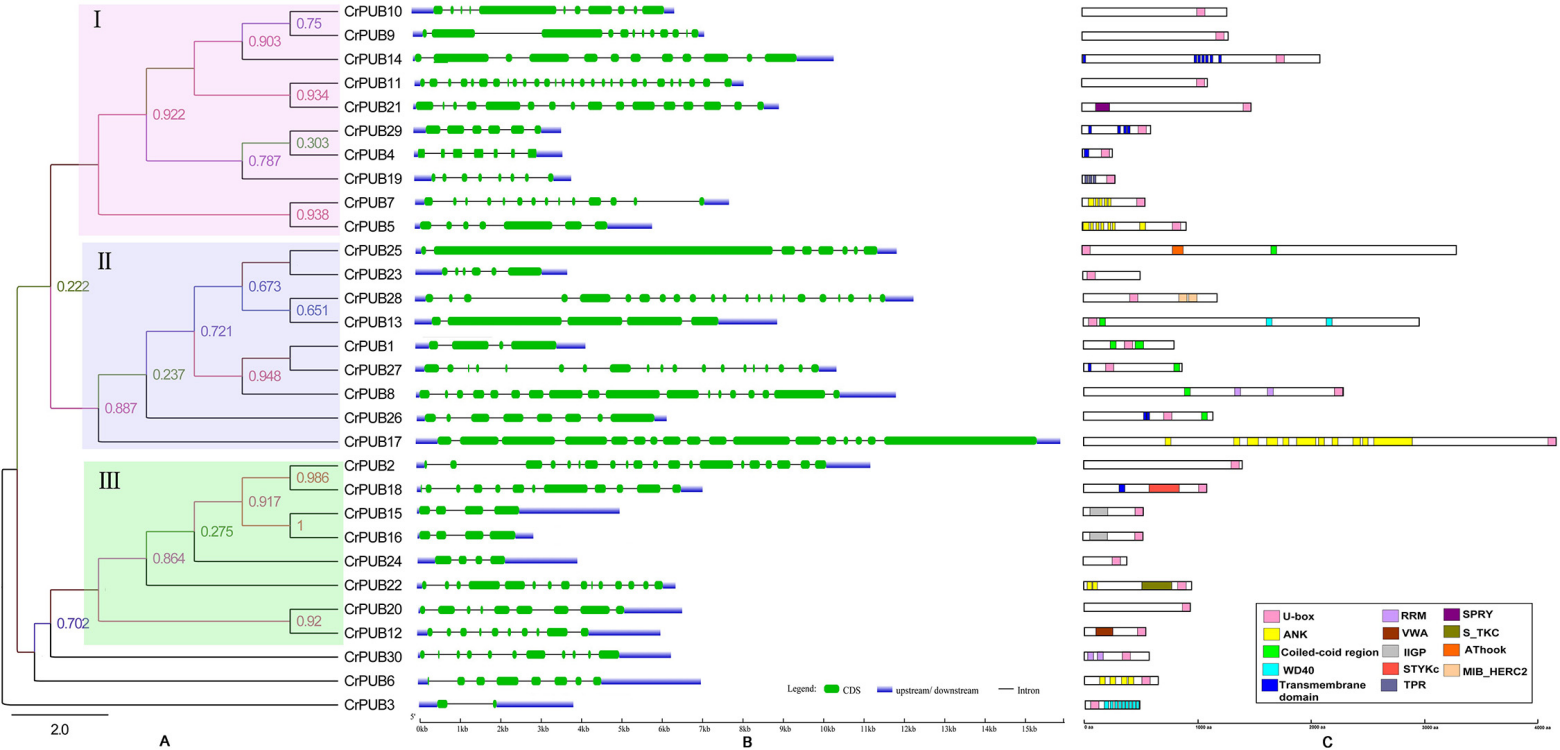
An analytical view of the CrPUB gene family. A. An unrooted tree summarizing the evolutionary relationships among the 30 members of the CrPUB family. Multiple alignments of the 30 PUB protein sequences from *C. reinhardtii* were conducted using ClustalX 2.1. The phylogenetic tree was constructed using PhyML3.01 and the ML method with 1,000 bootstrap replicates. The numbers on each node are Shimodaira-Hasegawa-like test indices of statistical support provided by PhyML. Bar = 2.0 is a branch length that represents the number of amino acid substitutions per site. The tree shows the 3 phylogenetic subfamilies (numbered I to III and marked with different color backgrounds) with high predictive values. B. Intron/exon structure: The gene structures were drawn using the online tool GSDS. As shown in the legend, the exons and introns are indicated by green rectangles and thin lines, respectively. The untranslated regions (UTRs) are indicated by blue boxes. The sizes of exons and introns can be estimated using the scale shown at the bottom. C. Schematic representation of the conserved motifs in the 30 CrPUB proteins elucidated using SMART and PROSITE online. The different domains are indicated by different colored boxes denoted at the bottom right corner. The lengths of the proteins and motifs can be estimated using the scale shown at the bottom.
