# Supplementary material for: Genome-Wide Survey and Expression Analysis of Chlamydomonas reinhardtii U-box E3 Ubiquitin Ligases (CrPUBs) Reveal a Functional Lipid Metabolism Module
Source: PLoS One. 2015 Mar 30;10(3):e0122600. doi: 10.1371/journal.pone.0122600 (PMC4378952; doi:10.1371/journal.pone.0122600)
Supplement: S1 Table — (PDF) [file pone.0122600.s001.pdf]

S1 Table

Gene name	Primer sequences	Gene name	Primer sequences
CrPUB1	F:5’-ACGTTGTCCGGGCGCATCA-3’	CrPUB16	F:5’-GTTCCTGTGGGACTTGCC-3’
	R:5’-AGCGCAGGTTGTGGTTGG-3’		R:5’-CGCCTCGTCTGCTTTGTT-3’
CrPUB2	F:5’-CAACCGCACTCGCCTGTA-3’	CrPUB17	F:5’-CTTCCGCACAAGCAGCAA-3’
	R:5’-GGCTCGCTCGTATGTGAA-3’		R:5’-ACCCGCCAGTAGCGATGA-3’
CrPUB3	F:5’-GAGGAGCTGGAGCCCGAT-3’	CrPUB18	F:5’-GCTCTGACGTGGCCTTTG-3’
	R:5’-GCAGCCGCAACAGGAACA-3’		R:5’-ATGGACGGCATGAACCCT-3’
CrPUB4	F:5’-ACTCAACCCGTCCCATCA-3’	CrPUB19	F:5’-CAAGGTCGTGGTGAGTTA-3’
	R:5’-AGCCGCAACAGGAACAGC-3’		R:5’-GCGAAATACTGGCGTAAA-3’
CrPUB5	F:5’-GACGGCGGAGGAGATGGT-3’	CrPUB20	F:5’-GCCCTTCTCCCTTCCCTC-3’
	R:5’-CTTTGAGGGCGTTGTTGG-3’		R:5’-ATGGCGGCTCGCTCGTAG-3’
CrPUB6	F:5’-TGCCCCATCACCCAGGAG-3’	CrPUB21	F:5’-CGATGAAGCGTCACCAGA-3’
	R:5’-CGTCCCACCGCGTTATTC-3’		R:5’-CACGGAGCTGAAGGAGGA-3’
CrPUB7	F:5’-TCCCTCGCTACCCACAGT-3’	CrPUB22	F:5’-CTCAAGCCCGCCAACATC-3’
	R:5’-GGCCTCCTCCTCCGCTTT-3’		R:5’-CCGACTCCACCACAGACA-3’
CrPUB8	F:5’-TGGACGAGCAGGTGACGA-3’	CrPUB23	F:5’-CTGGGCTTGGGTGGTGGA-3’
	R:5’-CCAGCAGGTTCCCAAACG-3’		R:5’-GCGGTGTTGGCAGTAGCA-3’
CrPUB9	F:5’-GGGTCGTGCAGTTGGTCT-3’	CrPUB24	F:5’-GCAGCAATGGTGTCGTCG-3’
	R:5’-CCCACAGTGATTCGCAGA-3’		R:5’-CCGCTGGGCAATCTTCTC-3’
CrPUB10	F:5’-CGCCTGGTGGCTGTCTTC-3’	CrPUB25	F:5’-GCGCATCAGCATCCAAGC-3’
	R:5’-CCTTCGCCGTCATCATCG-3’		R:5’-TTCCGTAACCGCGAACCC-3’
CrPUB11	F:5’-CCATCCTTAGCACCATCA-3’	CrPUB26	F:5’-TCGCCTACGACACCACCT-3’
	R:5’-TCCGTCCCTTCCGTTTCC-3’		R:5’-CAGCCCAGTCACCAACAC-3’
CrPUB12	F:5’-CAAGTCAACACCGGAAA-3’	CrPUB27	F:5’-GGACGCCGATGTGGAAGA-3’
	R:5’-TGCCACAATGAGGTTCG-3’		R:5’-TGTGGCCGCCAGAATCAC-3’
CrPUB13	F:5’-TCCACCCGCACATTCACA-3’	CrPUB28	F:5’-GCCGACTGCGAGTGGATT-3’
	R:5’-TGCTTCCCACAGCCACAG-3’		R:5’-GCCGGTGCAGATGATGAA-3’
CrPUB14	F:5’-CCTGCCGCCCGAGATGCT-3’	CrPUB29	F:5’-GGGATGCTGGATATTGGC-3’
	R:5’-CGCCGTGGTGTTTGATGC-3’		R:5’-CACCTCCTCCGGCGACAC-3’
CrPUB15	F:5’-TGCCCAATCACGCAGGAC-3’	CrPUB30	F:5’-AGTGGCGGTTTGGTCT-3’
	R:5’-AATCCGCTGCCGCTGGTA-3’		R:5’-CTGTTGGCGTGCTTGA-3’
