# Supplementary material for: Genome-Wide Survey and Expression Analysis of Chlamydomonas reinhardtii U-box E3 Ubiquitin Ligases (CrPUBs) Reveal a Functional Lipid Metabolism Module
Source: PLoS One. 2015 Mar 30;10(3):e0122600. doi: 10.1371/journal.pone.0122600 (PMC4378952; doi:10.1371/journal.pone.0122600)
Supplement: S2 Table — A: The primers used for the amplification of RNAi expression fragments from non-conserved domain-encoding regions. B: Primers randomized in design for the amplification of RNAi expression fragments. The sequence, length, Tm, GC% and product sizes of the primers are shown in the table. (PDF) [file pone.0122600.s002.pdf]

S2 Table

A					
Gene name	Primer sequences	Length	Tm (℃)	GC%	Product size
CrPUB5	F:5’-CGAGGAGGACGACGACGACA-3’	20bp	64.0	65.0	491bp
	R:5’-TGAGCGGGCACAGGAAGCAG-3’	20bp	66.7	65.0	
CrPUB11	F:5’-CGGTCATCAGCACCCTCAAC-3’	20bp	60.8	60.0	436bp
	R:5’-ATCTCCTCGTCCTCCGCCTC-3’	20bp	62.8	65.0	
CrPUB14	F:5’-TTCCTGCCGCCCGAGATGCT-3’	20bp	69.5	65.0	306bp
	R:5’-GCCGCCGTGGTGTTTGATGC-3’	20bp	68.8	65.0	
CrPUB23	F:5’-GAGCGAGCTGTGCAGGGTTG-3’	20bp	63.9	65.0	493bp
	R:5’-CGAAGCGTGCAGGTCGGTAG-3’	20bp	64.0	65.0	
CrPUB28	F:5’-AGCCGACTGCGAGTGGATTG-3’	20bp	63.3	60.0	405bp
	R:5’-GCGTTGCACTGTGCCTGACC-3’	20bp	64.5	65.0	
B					
Gene name	Primer sequences	Length	Tm (℃)	GC%	Product size
CrPUB5	F:5’-CGCCGTGTCCAACACCTTTG-3’	20bp	64.4	60.0	328bp
	R:5’-ACCCGTGTCAGCGAACTTGC-3’	20bp	63.2	60.0	
CrPUB11	F:5’-CCGTGAAGATGGTCGTGGAG-3’	20bp	60.9	60.0	422bp
	R:5’-CATGTGGCCCTTCGTGGTCT-3’	20bp	62.8	60.0	
CrPUB14	F:5’-CAAGAGGGCGTCGAAGGTGG-3’	20bp	65.1	65.0	441bp
	R:5’-GCGCAGGTTGTCGTTGTGGA-3’	20bp	64.9	60.0	
CrPUB23	F:5’-GCTGCCTGAGGAGCTGGTGA -3’	20bp	63.4	65.0	436bp
	R:5’-ACTGCCGCTGAAACGGTGGG -3’	20bp	67.6	65.0	
CrPUB28	F:5’- CTTCAGCGACGCCAAGGACG -3’	20bp	66.2	65.0	376bp
	R:5’-GCCAATCAGCTCGGCACAGG-3’	20bp	66.0	65.0	
